# A cross-sectional study on socio-demographic correlates of self-reported self-care practices for hypertension and type 2 diabetes among adults living in rural Kenya

**DOI:** 10.1186/s12913-024-12088-4

**Published:** 2024-12-19

**Authors:** MacKenna Schwarz, Bishal Gyawali, Dorothy Mwari Nkonge-Ngumba, Sylvia Khamati Anekha, Miriam Ngure, Tania Aase Dræbel

**Affiliations:** 1 Department of Public Health, Global Health Section, University of Copenhagen, Copenhagen, Denmark; 2 London School of Hygiene and Tropical Medicine, London, UK; 3 Danish Red Cross, Nairobi, Kenya; 4 Kenya Red Cross Society, Nairobi, Kenya

**Keywords:** Healthcare services, Self-care practices, Sociodemographic factors, Hypertension, Type 2 diabetes, Non-communicable diseases, Rural Kenya

## Abstract

**Background:**

Hypertension and type 2 diabetes are among the most common non-communicable diseases that contribute to a large number of adult morbidity and mortality in Kenya. The impact of these conditions may pose great challenges in rural areas with limited access to healthcare services. The objective of the study was to assess socio-demographic factors associated with self-reported self-care practices for hypertension and type 2 diabetes among adults living in rural Kenya.

**Methods:**

This study used data from the 2019 Baseline Assessment of the Prevention and Control of Non-Communicable Disease Project in Imenti South, Meru County conducted by the Kenyan Red Cross Society. A community-based study using a cross-sectional design was conducted among four hundred and thirty-five participants in Imenti South sub-County, Meru County in Kenya in November 2019. Chi-square test and logistic regression analyses were conducted to explore sociodemographic factors associated with self-reported self-care practices for hypertension and type 2 diabetes. Crude and Adjusted Odds Ratios with a 95% Confidence Interval (CI) were reported.

**Results:**

Among the 435 participants, 37.0% self-reported hypertension, while 15.4% reported having type 2 diabetes. Variances in self-care practices were evident between the conditions, notably in terms of adequate fruit and vegetable intake and blood pressure screening. Among individuals with type 2 diabetes, 94% lacked sufficient fruit and vegetable consumption, contrasting with 98.7% among hypertensive participants (*p* = 0.042). Similarly, a significant majority of individuals with hypertension (71.4%) had blood pressure screening (*p* = 0.031). Multivariable logistic regression analysis revealed that individuals over 40 years exhibited higher odds of good self-care practice for hypertension compared to their younger counterparts (AOR: 4.67, 95% CI: 1.53–14.27, *p* = 0.007), whereas those residing in Mitunguu were 71% less likely to engage in such practices than those in Abogeta (AOR: 0.29, 95% CI: 0.09–0.90, *p* = 0.033). However, none of the variables demonstrated a significant association with self-reported self-care practices for type 2 diabetes following adjustment for potential confounding variables in the multivariable logistic regression analysis.

**Conclusions:**

Our study identified socio-demographic factors, including age (> 40 years) and ward (Mitunguu), associated with self-reported self-care practices for hypertension among adults living in rural Kenya. However, we did not find significant associations between sociodemographic factors and self-care practices for type 2 diabetes. Furthermore, factors such as gender, education level, marital status, religion, employment status, and average monthly income did not show significant associations with self-care practices for hypertension or type 2 diabetes. These results provide insights regarding sociodemographic factors associated with self-care practices for hypertension among adults living in rural Kenya. Our study underscores the relevance of considering socio-demographic factors when making evidence-based policy decisions and designing intervention strategies tailored to the adult population in rural Kenya.

**Supplementary Information:**

The online version contains supplementary material available at 10.1186/s12913-024-12088-4.

## Introduction

Kenya is experiencing an epidemiological transition, characterized by a shift from a high burden of communicable diseases to Non-Communicable Diseases (NCDs) [1]. Concurrently, this poses an additional disease burden on Kenya, which is still struggling with preventing and controlling common communicable diseases, such as malaria, HIV and AIDS, and tuberculosis among others [2]. Since 2014, the percentage of deaths attributed to NCDs in Kenya has increased from 27 to 39% [1]. It is estimated that hypertension and type 2 diabetes account for 24% and 5%, respectively of these deaths in Kenya [3]. The increase in hypertension and type 2 diabetes is associated with demographic, epidemiological, socioeconomic, and nutritional transitions, resulting from globalization and urbanization [4].

Increased research on NCDs in recent decades has shed light on the prevalence of hypertension and type 2 diabetes in rural and urban areas of Kenya. The current prevalence of hypertension in Kenya is 28.7% [5]. In 2013, the prevalence of diabetes was estimated to 3,6% with a projected increase to 4,4% by 2035 [6]. According to the 2015 Kenya STEPwise surveyor NCDs, 97% of the Kenyan adult population are exposed to one of the four major risk factors (i.e., tobacco, alcohol, unhealthy diets, and lack of physical activity) and 25% are exposed to three or more [3]. Additionally, it has been noted that there is a lack of access to medication and health services in rural Kenya, where a majority of the population lives [7]. Moreover, data on self-care practices for NCDs and sociodemographic factors that affect these practices are lacking in rural SSA [8] and Kenya [9]. The Kenyan National NCD strategy aims to reduce premature mortality in Kenya from 39 to 30% by 2025 through sectoral and multisectoral coordination and governance, minimizing exposure to risk factors, health system response, advocacy and community mobilization, and surveillance, monitoring, and research [1]. In line with this strategy, the Kenyan Ministry of Health has made the commitment to increase the community and institutional screening of NCDs and risk factors, access to quality care and improvement of treatment outcomes, as outlined in the National Strategic Plan for NCD prevention and control [1]. In spite of this, progress has been slow and uneven.

Populations living in socioeconomically disadvantaged neighbourhoods are less likely to have a usual source of care and more likely to have unmet medical needs [10–14]. Financial costs and geographical distance can limit access to healthcare, and influence self-care practices, especially for people living with type 2 diabetes and hypertension [15]. In Kenya, health services are delivered by a mix of public and private entities including the Kenyan Ministry of Health, faith-based organizations and private-for-profit organizations. Services for prevention, treatment and control of type 2 diabetes and hypertension is accessible at the six levels of the Kenyan health system [2]. At the 1st level, Community Health Services provide basic preventative and curative services for minor ailments at the community and household level [1]. At the 2nd level, dispensaries act similarly to Health Centres, though they do not have in-patient facilities and capacities [1]. The 3rd level consists of Health Centres, which function as small hospitals, but with reduced facilities and capacities. At the 4th level, Sub-County Hospitals function as a hospital that can provide comprehensive care and surgical services, in addition to being able to act as a referral centre for patients. The 5th level includes County Referral Hospitals, previously called provincial hospitals, which provide similar services to level 4, in addition to conducting research and other specialized care. At the 6th level, National Referral Hospitals, which are also teaching, and research hospitals often specialize in specific treatments. These hospitals are also available to other Eastern and Central African countries. Care for diabetes is usually offered through specialized clinics in public hospitals at levels 4 to 6 [1, 2]. In some counties, health centres and dispensaries also provide medication to patients, but this is not standard procedure [1, 2]. Patients move from one level of the system to another using a referral letter.

Based on the current literature, it is evident that there are many factors that come into play in self-care practices for hypertension and type 2 diabetes, including age, gender, level of education, marital status, employment status, religion and average monthly household income [16, 17]. Self-care practices are also influenced by other factors, such as costs, location (access), healthcare services sought and social support [18–20]. As for costs, there are significant out-of-pocket costs associated with treatment and management of type 2 diabetes and hypertension in public facilities in Kenya [21]. The higher the level of healthcare facility the more likely the cost is going to be high [22]. Hence, a person’s ability to afford these costs, whether through private medical insurance or national insurance, significantly influence their self-care practices [21, 22]. As to location (access), e.g. the healthcare provided at the level 6 health care facility in Kenya, would mainly be in the capital centres as these are the main referral facilities with specialized healthcare workers and services. In relation to healthcare services sought, some services are only provided at higher levels of the system, e.g. type 2 diabetes and hypertension treatment may not be available in all facilities at level 1–3 [1, 2]. Further, one study conducted in rural Nigeria indicated that wealth was a key determinant of access to healthcare [15], which may also impact self-care practices [21, 22].

Women and poorer individuals and communities are more likely to forego formalized healthcare due to high costs and limited accessibility of services [15]. Research on the topic, however, lacks consensus, with variations in findings likely due to differing social structures and contexts across studies [23, 24]. Gender dynamics, social positions and roles of women are social constructs varying over time and place. For instance, Opare-Addo et al. [25], Rasul et al. [26] and Hjelm and Atwine [27] have established gender differences in the utilization of services for chronic illnesses. Janssens et al. [15] and Mwangi et al. [16] found that women and economically deprived individuals were more likely to forego formalized healthcare due to high costs and limited accessibility of services. Social position and gender dynamics are closely related to women’s health and access to care, influencing their self-care practices, whether through personal autonomy, or household decision-making. When women do exercise autonomy in healthcare choices, high costs often play a crucial role, as highlighted by Janssens et al. [15]. In contrast, Do et al. have not established an association between gender and self-care practices [28]. Research on the association of marital status and self-care practices for hypertension, seem to suggest that married individuals or those living with relatives or within larger households may have more social resources to engage in self-care [18, 19]. Patients who are separated or divorced are less likely to be on treatment as these may dispose of less social resources to practise self-care [20]. Previous studies have assessed the association between educational attainment and self-care practices for type 2 diabetes A study by Jansen, Rademakers and Heijmans [29] has shown that higher educational attainment is associated with higher scores on the health literacy aspects, appraisal of health information and navigating the healthcare system. Similarly, a study by Noppert et al. [30] reports that educational attainment is a predictor for cardiometabolic health, even in young adults. In a study among individuals with type 2 diabetes, Alguwaihes et al. [31] report an association between educational attainment and healthcare utilization and “Self-Care Behavior”. The association between educational attainment and self-care practices is also confirmed in a study among Mexicans, Mexican Americans and US-Mexico migrants [32]. Galeema et al. [33] show that patients with lower educational attainment are less likely to adhere to cardiac rehabilitation than their counterparts with higher educational attainment and may not benefit to the same extent from cardiac rehabilitation.

Even if health services for hypertension and type 2 diabetes are available and accessible, a significant number of people living with hypertension and type 2 diabetes are at increased risk of complications of hypertension and type 2 diabetes because of inadequate self-care practices. Self-care practices, such as maintaining a healthy diet, engaging in physical activities, avoiding alcohol and smoking are essential for control of blood sugar level and blood pressure. Reducing risks of hypertension and complications from diabetes requires a daily effort, which can become challenging for persons living with hypertension and type 2 diabetes [34]. A key characteristic of living with NCDs is the individual responsibility for self-care practices placed on the patient. In a context of scarce resources, patients are to a very large extent dependent on their family resources and their own capabilities to successfully manage and control their hypertension and type 2 diabetes. Patients’ management of hypertension and type 2 diabetes largely depends upon the resources at their disposal, whether economic or social, yet it depends just as much on the patient’s capability of mobilizing the right kind of resources that will help them to for example access diabetes health services or adjust diet. However, little is known about factors associated to patients’ self-care practices for hypertension and type 2 diabetes, especially in low-income countries [8].

While the influence of sociodemographic factors on self-care practices to for NCDs is widely documented in the Northern hemisphere and Asia [4, 5], little is known about this in the context of rural Kenya [35]. Without such insight, health policy, programmes, and planning may fail to adequately address the risk factors for poor NCD prevention and control as well as miss opportunities to enhance the appropriate use of health services. Furthermore, morbidity and mortality may increase, and already existing health inequalities may be further exacerbated. Identification of sociodemographic factors associated with self-care practices will facilitate the development of new interventions and reduce barriers to accessing care that are associated with peoples’ socioeconomic situation and demographic characteristics. The purpose of this study was to assess sociodemographic factors associated with the self-reported self-care practices for hypertension and type 2 diabetes among adults living in rural Kenya.

## Methods

### Data source, study design and setting

This study used data from the 2019 Baseline Assessment of the Prevention and Control of NCDs (hypertension and type 2 diabetes) Project in Imenti South, Meru County conducted by the Kenya Red Cross Society [36]. The study was a community-based cross-sectional survey conducted from 1st to 30th November 2019 in Imenti South sub county, Meru County, Kenya. Imenti South is situated approximately 274 kms northeast of Nairobi and just east of Mount Kenya. Similar to other regions in Eastern Africa, Meru County is experiencing an epidemiological transition, as the burden of NCDs is replacing communicable diseases as the leading causes of morbidity and mortality [1]. Hypertension and type 2 diabetes are key contributors to this and in 2018, the county has 60,275 cases in its adult population enrolled in type 2 diabetes and hypertension treatment clinics [37]. Of these cases, Imenti South recorded 4,296 diabetes-related morbidities and 11,800 cases of hypertension in 2018 [37]. The population of Meru County is approximately 1,545,714, with approximately 767,698 males, 777,975 females, and 41 transgender [38]. There are 426,360 households in Meru County with an average household size of 3.6 persons [38]. Meru County is largely rural and it is made up of 9 sub-counties, including Imenti South, which is where this survey was conducted [36]. Imenti South consists of eight wards, five of which were included in this study (Mitunguu, Abogeta East, Abogeta West, Igoji East, and Igoji West). Kenya is divided by counties, followed by sub-counties, and then by ward, which is where villages and households are found. A ward is the term used to describe the smallest administrative unit in a sub-county, where people reside [38].

### Study population and participants

The study population was all adults aged 18 years and above, who were members of sampled households and were within the households during the time of the interview. All the household heads or their spouses in the sampled wards were randomly sampled and recruited for the survey.

### Sample size and sampling technique

To determine the sample size, Yamane formulae (1967) for sample size calculation was used. Sample size n = N/1 + N(e2) Where, N = was the estimated number of households, which was 111,591. This was estimated from the population Size of 458,362 [38], given a household size of 4, while was 0.5 the margin of error. The sample size selected was thus 398, with an additional 10% contingency for non-response, resulting in a final sample size of 437 households for the assessment. In the actual survey, a total of 435 respondents were interviewed with the household questionnaire. A multistage sampling technique was applied to recruit the participants. Imenti South Sub Country has eight administrative wards [38]; five of the eight wards were initially selected randomly. Additionally, in each of these five wards, 10% of villages were randomly selected, and these were the areas that were surveyed. Each village was then allocated a sample size proportional to the total households in each village. The first households were selected from each village by spinning a bottle, where the tip of the bottle was pointed. The teams then systematically interviewed households “as the eagle fly” i.e. on a straight line and determined the interval by dividing their allocated sample size by the estimated number of households on the interview path. In the event they reached the end of the cluster before concluding their sample to interview, they took the right turn and continued interviewing with the same skip interval. Ineligible households were replaced by the immediate next households, after which the skip pattern was maintained based on the original pattern. When reaching the household, the head of household or, in their absence, their spouses were asked for informed consent to participate in the study. Once the head of household or, in their absence, their spouses had given their consent, they were interviewed. Households were ineligible if the head of household, or spouses of the head of household was not at home at the time of the study. Pregnant women and persons below the age of 18 were not interviewed. Verbal informed consent was obtained from all participants for inclusion in the study. Participants who did not provide informed verbal consent or were not able to complete the questionnaire were excluded.

### Measures

The dependent (outcome) variables of the study were self-reported self-care practices for hypertension and type 2 diabetes. These variables were categorised into binary outcomes (good vs poor) for both conditions. This categorisation was determined based on several questions, including whether participants used tobacco, consumed alcohol, had increased physical activity, had increased fruit and vegetable intake, had their blood pressure and blood glucose checked, were currently taking hypertensive and diabetic medications, and regularly monitored their blood pressure and blood glucose at home. The independent variables were socio-demographic variables, including age group in years (< = 40, > 40), gender (male, female), ward (Abogeta, Igoji, Mitunguu), education level (primary level or lower, secondary level or upper), marital status (unmarried, married), religion (Christian (catholic), Christian (protestant)), employment status (unemployed, employed), average monthly income (< 3,000 Kenyan Shillings (KsH) or < 24 US Dollars (1 KsH = 0.0083 US Dollar, September 2022), 3,000–6,000 KsH or 24–49 US Dollars, > 6,000 KsH or > 49 US Dollars).

### Study measurements

Self-care practices were assessed using a questionnaire comprising 7 inquiries both for hypertension and type 2 diabetes, each requiring “Yes”, or “No” responses. The questionnaire was developed for this study (supplementary material). A score of “1” was assigned for “Yes” responses, and “0” for “No” responses. Then, a score equal to or greater than the mean response was classified as indicating good self-care practice, while scores below the mean were considered indicative of a poor level of practice. Hypertension and type 2 diabetes were self-reported.

Data on current smoking was obtained by asking participants, “Do you currently smoke any tobacco products, such as cigarettes?” with options for “Yes” or “No” reponses. Alcohol consumption was assessed by participants indicating “Yes” or “No” in response to the question, “Do you currently consume alcoholic drinks, such as beer, wine, busaa, whiskey, chang’aa?”.

The level of physical activity was determined by querying participants about the number of days and time spent on vigorous and/or moderate activities related to work, travel, and leisure. The number of Metabolic Equivalent of Task (MET) minutes per week was calculated using the standard formula WHO STEPS and categorized as low (< 600 MET minutes per week) or moderate/high (≥ 600 MET minutes per week) to determine if participants had increased physical activity.

Participants self-reported their fruit and vegetable consumption in a typical week. The number of servings of fruits and vegetables per day was calculated by summing the average intake per day and categorized into < 5 servings per day or ≥ 5 servings per day following WHO recommended standards to determine if participants had tried to increase their intake of fruits and vegetables.

Screening for blood pressure and blood glucose was assessed by asking participants if they had ever had their blood pressure and blood glucose checked. Medication adherence was determined by asking participants if they were currently taking hypertensive and diabetic drugs. Regular monitoring was assessed by asking participants if they regularly monitored their blood pressure and blood glucose at home.

### Data collection

The household questionnaire was uploaded onto portable electronic devices, which allowed the research assistants to interview and record responses [36]. Questionnaires were administered by face-to-face interviews. Interviews of participants were conducted by 25 trained research assistants in the selected villages. Research assistants were trained for a period of 3 days on key areas including key aspects of the study protocol, data collection tools, the background to the study, and research ethics including consenting, interviewing skills, and pretesting. They were also trained on sensitivities around interviewing the target groups. The questionnaire included two sections: the first section captured participants’ sociodemographic information and the second section, captured participant’s self-reported data on hypertension and type 2 diabetes as well as their selected behavioural risk factors. The questionnaire was pretested on 2% of the study participants found outside of the study area and modifications were made on the basis of the findings.

### Statistical analysis

Statistical analyses were conducted using Stata version 16 (Stata Corp, College Station, TX, USA). Descriptive statistics were used to summarize the data by frequency and percentages. Comparative analyses between individuals with hypertension, type 2 diabetes and healthy individuals were conducted using the Chi-square test to assess differences in socio-demographic characteristics and self-care practices among individuals with hypertension and type 2 diabetes only.

The association between self-care practices and socio**-**demographic factors was first identified by bivariable analysis using binary logistic regression. Those variables with *p-*value of < 0.25 in the bivariable logistic regression analysis were identified as candidate variables for the final model, multivariable binary logistic regression to control the potential effect of confounding variables. Both the crude odds ratio (COR) and adjusted odds ratio (AOR) with 95% confidence intervals (CIs) were estimated to show the strength of associations. Finally, a *p*-value of < 0.05 in the multivariable logistic regression analysis was used. For this study, the Hosmer and Lemeshow goodness-of-fit test was used to assess whether the necessary assumptions for the application of multiple logistic regression were fulfilled and the variance inflation factor was used to check for multicollinearity among selected independent variables. The entire analysis was guided by Tusubira et al. [8].

## Results

### Sociodemographic characteristics

The mean age of the participants was 49.1 years (SD: ± 17.6). The majority were female (60.5%, 263/435), while 39.8% (173/435) resided in Mitunguu. Most participants were married (65.1%, 283/435) and employed (90.3%, 393/435). Additionally, 70.8% (308/435) had primary or lower levels of education, and 64.8% (282/435) identified as Christian (Protestant). Nearly half of the participants (47.1%, 205/435) reported a low level of average monthly income. Among the participants, 37.0% (161/435) self-reported hypertension, while only 15.4% (67/435) reported having type 2 diabetes.

Significant variations were observed in the self-reported prevalence of hypertension and type 2 diabetes across different wards (*p* < 0.0001), marital status (*p* = 0.008), religion (*p* = 0.011), employment status (*p* = 0.011), and average monthly income (*p* < 0.0001). While over half of individuals with hypertension (57.1%, 62/161) resided in Mitunguu, all individuals living with type 2 diabetes (67/67) were also residents of this region. In contrast, only 6.8% (14/207) of the healthy population resided there. Despite a higher number of married individuals across all conditions, marriage comprised 71% (144/207) of healthy participants, compared to 68.7% (46/67) of individuals with type 2 diabetes and 55.9% (90/161) of hypertensives. Similarly, although a greater number of individuals identified as Christian (Protestant) across all conditions, Christian (Protestant) individuals constituted nearly three-quarters (77.6%, 52/67) of those with diabetes, compared to 67.7% (109/161) of individuals with hypertension and 58.5% (121/207) of healthy individuals. Additionally, employment rates were higher across all conditions, with 98.5% (66/67) of individuals with diabetes being employed, compared to 90.8% (188/207) of healthy individuals and 86.3% (139/161) of hypertensives. Furthermore, a significant proportion of participants with type 2 diabetes (59.7%, 40/67) and healthy individuals (57.0%, 118/207) had a low average monthly income, compared to 29.2% (47/161) of patients with hypertension (Table 1).

**Table 1:** Socio-demographic characteristics of members of households

Variables	HTN *N* = 161	DM *N* = 67	Healthy individuals *N* = 207	*p*-value
Age (years) *n* (%)				0.268
< = 40	69 (42.8)	22 (32.8)	75 (36.2)	
> 40	92 (57.2)	45 (67.2)	132 (63.8)	
Gender *n* (%)				0.914
Female	99 (61.5)	41 (61.2)	123 (59.4)	
Male	62 (38.5)	26 (38.8)	84 (40.6)	
Ward *n* (%)				< 0.0001*
Abogeta	66 (41.0)	0	55 (26.5)	
Igoji	3 (1.9)	0	138 (66.7)	
Mitunguu	92 (57.1)	67 (100.0)	14 (6.8)	
Education level *n* (%)				0.991
Primary level or lower	114 (70.8)	47 (70.2)	147 (71.0)	
Secondary level or upper	47 (29.2)	20 (29.8)	60 (29.0)	
Marital Status *n* (%)				0.008*
Unmarried	71 (44.1)	21 (31.3)	60 (29.0)	
Married	90 (55.9)	46 (68.7)	147 (71.0)	
Religion *n* (%)				0.011*
Christian (catholic)	52 (32.3)	15 (22.4)	86 (41.5)	
Christian (protestant)	109 (67.7)	52 (77.6)	121 (58.5)	
Employment status *n* (%)				0.011*#
Unemployed	22 (13.7)	1 (1.5)	19 (9.2)	
Employed	139 (86.3)	66 (98.5)	188 (90.8)	
Average Monthly Income in USD *n* (%)				< 0.0001*
Low (< $24)	47 (29.2)	40 (59.7)	118 (57.0)	
Middle (~ $24–49)	57 (35.4)	14 (20.9)	45 (21.7)	
High (> $49)	57 (35.4)	13 (19.4)	44 (21.3)	

*Acronyms*: *HTN* hypertension, *DM* diabetes mellitus, *$* United States Dollar, # Fisher’s exact

^*^P-value < 0.05

### Self-care practices for hypertension and type 2 diabetes

Differences in self-care practice components were observed between the conditions, particularly in adequate fruits and vegetable intake and blood pressure screening. Among participants with type 2 diabetes, 94% (63/67) lacked adequate fruit and vegetable intake, while this percentage was 98.7% (159/161) among hypertensive participants (*p* = 0.042). Similarly, a majority of hypertensive patients (71.4%; 115/161) underwent blood pressure screening, showing a significant difference (*p* = 0.031) (Table 2).

**Table 2:** Self-care practices among individuals with HTN and DM

Variables	HTN *N* = 161	DM *N* = 67	*p*-value
Adequate fruits and vegetable intake *n* (%)			0.042*
No	159 (98.7)	63 (94.0)	
Yes	2 (1.2)	4 (6.0)	
Current smoking *n* (%)			0.620
No	125 (77.6)	54 (80.6)	
Yes	36 (22.4)	13 (19.4)	
Current alcohol use *n* (%)			0.414
No	114 (70.8)	51 (76.1)	
Yes	47 (29.2)	16 (23.9)	
Adequate physical activity *n* (%)			0.280
No	34 (21.1)	10 (14.9)	
Yes	127 (78.9)	57 (85.1)	
Blood pressure screening *n* (%)			0.031*
No	46 (28.6)	NA	
Yes	115 (71.4)	NA	
HTN medication adherence (*n* = 63 (%))			0.757
No	36 (57.1)	NA	
Yes	27 (42.9)	NA	
Blood pressure monitoring *n* (%)			0.100
No	110 (68.3)	NA	
Yes	51 (31.7)	NA	
Blood glucose screening *n* (%)			0.159
No	NA	52 (77.6)	
Yes	NA	15 (22.4)	
DM medication adherence (*n* = 10 (%))			0.116
No	NA	5 (50.0)	
Yes	NA	5 (50.0)	
Blood glucose monitoring* n* (%)			0.641
No	NA	66 (98.5)	
Yes	NA	1 (1.5)	

*Acronyms*: *HTN* hypertension, *DM* diabetes mellitus

^*^P-value < 0.05

### Factors associated with hypertension and type 2 diabetes self-care practices

Factors associated with self-care practices in hypertension were explored through bivariable logistic regression, revealing significant associations between various factors, such as age, gender, ward, marital status, religion, education, and monthly income and hypertension self-care practice (Table 4). Similarly, ward and religion were found to have significant associations with diabetes self-care practice (Table 3). However, upon adjusting for potential confounding variables in multivariable logistic regression, only age and ward remained significantly associated with good self-care practice for hypertension at a *p*-value < 0.05. Specifically, individuals older than 40 years had increased odds of good self-care practice for hypertension compared to those aged 40 years or younger (AOR: 4.67, 95% CI: 1.53–14.27, *p* = 0.007), while participants residing in Mitunguu were 71% less likely to engage in good self-care practice compared to those in Abogeta (AOR: 0.29, 95% CI: 0.09–0.90, *p* = 0.033) (Table 3). Conversely, none of the variables showed a significant association with self-care practice for type 2 diabetes after adjusting for potential confounding variables in the multivariable logistic regression analysis at *p*-value < 0.05 (Table 4).

**Table 3:** Bivariable and multivariable analysis of socio-demographic factors associated with self-care practice among HTN patients (*N* = 161)

Variables	Self-care practice among HTN patients			
	Good n (%)	Poor n (%)	COR (95% CI)	AOR (95%CI)
Age (years) *n* (%)				
< = 40	5 (10.2)	37 (33.0)	1	1
> 40	44 (89.8)	75 (67.0)	4.34 (1.58,11.86)	4.67 (1.53, 14.27)*
Gender *n* (%)				
Female	30 (61.2)	81 (72.3)	1	1
Male	19 (38.8)	31 (27.7)	1.65 (0.81, 3.35)	1.12 (0.48, 2.63)
Ward *n* (%)				
Abogeta	15 (30.6)	26 (23.2)	1	1
Igoji	26 (53.1)	36 (32.1)	1.25 (0.5, 2.81)	1.05 (0.40, 2.74)
Mitunguu	8 (16.3)	50 (44.7)	0.27 (0.10, 0.73)	0.29 (0.09, 0.90)*
Education level *n* (%)				
Primary level or lower	39 (79.6)	76 (67.8)	1	1
Secondary level or upper	10 (20.4)	36 (32.2)	0.54 (0.24, 1.20)	0.65 (0.25, 1.66)
Marital Status *n* (%)				
Unmarried	14 (28.6)	46 (41.1)	1	1
Married	35 (71.4)	66 (58.9)	1.74 (0.84, 3.59)	1.82 (0.77, 4.34)
Religion *n* (%)				
Christian (catholic)	23 (46.9)	37 (33.0)	1	1
Christian (protestant)	26 (53.1)	75 (67.0)	0.55 (0.28, 1.01)	0.65 (0.25, 1.66)
Employment status *n* (%)				
Unemployed	3 (6.1)	8 (7.1)	1	-
Employed	46 (93.9)	104 (92.9)	1.17 (0.29, 4.64)	-
Average Monthly Income in USD *n* (%)				
Low (< $24)	30 (61.2)	55 (49.1)	1	1
Middle (~ $24–49)	9 (18.4)	29 (25.9)	0.56 (0.23, 1.35)	0.90 (0.33, 2.44)
High (> $49)	10 (20.4)	28 (25.0)	0.65 (0.28, 1.52)	0.85 (0.32, 2.23)

Adjusted for age, sex, ward, marital status, religion, education and monthly income

*COR* Crude Odds Ratio, *AOR* Adjusted Odds Ratio

^*^P-value < 0.05

**Table 4:** Bivariate and multivariable analysis of socio-demographic factors associated with self-care practice among DM patients (*N* = 67)

Variables	Self-care practice of DM patients			
	Good n (%)	Poor n (%)	COR (95% CI)	AOR (95%CI)
Age (years) *n* (%)				
< = 40	2 (6.7)	9 (24.3)	1	1
> 40	28 (93.3)	28 (75.7)	4.49 (0.89, 22.72)	3.65 (0.67, 19.90)
Gender *n* (%)				
Female	19 (63.3)	24 (64.8)	1	
Male	11 (36.7)	13 (35.2)	1.06 (0.39, 2.91)	
Ward *n* (%)				
Abogeta	9 (30.0)	4 (10.8)	1	1
Igoji	13 (43.3)	12 (32.4)	0.48 (0.11, 1.98)	0.88 (0.17, 4.46)
Mitunguu	8 (26.7)	21 (56.8)	0.16 (0.04, 0.70)	0.29 (0.06, 1.44)
Education level *n* (%)				
Primary level or lower	24 (80.0)	26 (70.3)	1	
Secondary level or upper	6 (20.0)	11 (29.7)	0.59 (0.18, 1.84)	
Marital Status *n* (%)				
Unmarried	10 (33.3)	12 (32.4)	1	
Married	20 (66.7)	25 (67.6)	0.96 (0.34, 2.67)	
Religion *n* (%)				
Christian (catholic)	15 (50.0)	8 (21.6)	1	1
Christian (protestant)	15 (50.0)	29 (78.4)	0.27 (0.09, 0.79)	0.35 (0.10,1.21)
Employment status *n* (%)				
Unemployed	1	0	-	
Employed	29	37	-	
Average Monthly Income in USD *n* (%)				
Low (< $24)	19 (63.3)	18 (48.6)	1	
Middle (~ $24–49)	3 (10.0)	6 (16.2)	0.47 (0.10, 2.18)	
High (> $49)	8 (26.7)	13 (35.2)	0.58 (0.19, 1.73)	

Adjusted for age, ward, and religion

*COR* Crude Odds Ratio, *AOR* Adjusted Odds Ratio

^*^P-value < 0.05

## Discussion

This cross-sectional study’s aim was to assess socio-demographic factors associated with self-reported self-care practices for hypertension and type 2 diabetes among adults living in rural Kenya. The results show that socio-demographic factors, including ward and age are significantly associated with self-care practices for hypertension among adults living in rural Kenya. Marital status, gender, education level, religion, employment status, and average monthly income were not significantly associated with self-care practices for hypertension or type 2 diabetes.

Our study shows that the ward is a factor associated with self-care practices for hypertension. Participants who live in Mitunguu are less likely to practise self-care for hypertension. This may serve to illustrate that among the rural population living in the Mitunguu ward, geographical distance to health facilities is a determinant of self-care practices for hypertension. It is widely documented that geographical distance between area of residence and health facilities is a factor in self-care practices. Acharya et al., [39] have shown that the level of self-care practices for hypertension were signifyingly lower among patients living in rural areas compared to self-care level of patients living in urban areas. Numerous other studies show that health services are much less likely to be used when located far from patients’ residences [40–43]. Factors determining self-care practices among patients with hypertension include built environment, health facilities’ location, availability and efficiency of transportation, walkability of route to health facility and level of walkable environment of health facilities that are located within walking distance and can be reached via a walkable environment. Moreover, high out-of-pocket costs related to transport for self-care practices has been identified as a barrier to access and use health services [21, 22]. Most primary healthcare facilities offer services at low or no cost to the patients [1, 2], which is why most people who are farmers (generally subsistence farmers with low income) use these facilities more. Thus, the affordability of transportation to health facilities is crucial for self-care practices among patients whose residence is located far from health facilities. Several studies highlight financial and transportation constraints as significant barriers to self-care practices for hypertension. For instance, Rahmawati and Bajorek found that patients with hypertension in rural areas often prefer easily available and inexpensive alternatives to hypertensive medicines, which are less accessible and affordable in comparison [44].

Age was also found to be a factor associated with self-care practices for hypertension, with individuals over 40 were significantly more likely to engage in self-care practice. This may be due to increased awareness and a greater need for ongoing treatment and monitoring among older adults. However, we have identified only a few other studies supporting this finding. Sivakumar et al. [45] found that respondents above 55 were more likely to practice self-care. Similarly, Dasgupta et al. [46] showed that patients above 60 were much more likely to engage in self-care for hypertension compared to those below 60 years. Warren-Findlow, Seymour and Huber [47] found that patients above 50 years old were more likely to practice hypertension self-care. In contrast, other studies indicate that the older the patients, the poorer the self-care practices [48, 49] or that age is not a predictor of self-care practices [40, 50, 51]. Another explanation for this result may be the study’s methodology and the characteristics of the study population, i.e. adults living in rural Kenya. In our study, age interacts with hypertension self-care practices and the severity of hypertension may worsen with age. Another plausible reason is that older individuals may have better resources to practice self-care.

We did not find significant associations between sociodemographic factors and self-care practices for type 2 diabetes. This finding contradicts that of Okafor et al., which reported a significant influence of socio-demographic characteristics on type 2 diabetes self-care practice [51]. A possible explanation for this discrepancy could be that individuals may not be well informed about the disease, may lack access to resources that support effective self-care, or may face high costs associated with treatment and care.

This study is one of the few that have assessed socio-demographic factors associated with self-care practices for hypertension or type 2 diabetes among adults in rural Kenya. The strengths of the study include random sampling of participants and a very high response rate. However, the study has several limitations. First, the design effect was not considered during sample size determination. Second, the cross-sectional nature of the survey limits our understanding of causal associations and reverse causality bias could be present; longitudinal studies are needed to strengthen our findings. Third, despite using multistage sampling, randomness cannot be guaranteed, especially with only a few wards selected. Fourth, the survey relied on self‐reports, which are subject to recall and social desirability bias. Fifth, the analysis did not consider the stages of hypertension and type 2 diabetes or whether the respondents have complications of hypertension or type 2 diabetes. Lastly, our study considered self-care practices as proxies, and did not include key domains such as weight management, low-salt diet, and foot care for both diabetes and hypertension due to data limitations. This limitation could potentially impact the interpretation of our results, and therefore, caution should be exercised when drawing conclusions.

Our study’s findings suggest that both ward and age should be considered in system and policy decisions related to hypertension self-care practices. Interventions addressing geographical distance between wards and health facilities, such as improving transportation affordability and efficiency in Mitungu ward, are likely to benefit hypertension self-care practices among residents. Additionally, age-group-based interventions targeting hypertension self-care at different life stages are likely to strengthen self-care practices among older and elderly adults.

Based on our study, the following five recommendations should be considered by policy-makers as follows:

1. High number of self-reported hypertension and type 2 diabetes, respectively 37% and 15.4%. Public health interventions for screening and early detection of hypertension and type 2 diabetes should be tailored to specific places.
2. Respectively 97% of participants with self-reported hypertension and 94% of participants with self-reported type 2 diabetes also report not eating sufficient fruits and vegetables. Health policy and system need to engage with community to increase knowledge on basic hypertensive self-care practices, i.e. diet.
3. Services for hypertension and type 2 diabetes are available, yet patients from Mitunguu ward are much less likely to practice self-care because of geographical distance to health facilities. Policy and system need to enhance affordability, accessibility and efficiency of transport between ward and health facilities located far from the population’s residence.
4. Participants less than 40 years were less likely to practice self-care than their older counterparts. We recommend interventions to increase community awareness about self-care practices for hypertension that factor in different needs of adults below 40 years.
5. Participants less than 40 and participants living in Mitunguu ward were less likely to practice self-care for hypertension. We recommend enhanced screening and early detection of hypertension among adults below 40 years and adults should be tailored to specific places.

## Conclusions

Our study assessed socio-demographic factors associated with self-care practices for hypertension and type 2 diabetes among adults living in rural Kenya. This study identified specific socio-demographic factors, including age (> 40 years) and ward (Mitunguu), that were associated with hypertension self-care practices. However, we did not find significant associations between sociodemographic factors and self-care practices for type 2 diabetes. Furthermore, factors such as marital status, gender, education level, marital status, religion, employment status, and average monthly income did not show significant associations with self-care practices for either hypertension or type 2 diabetes. These results provide insights regarding the socio-demographic factors associated with self-care practices for hypertension among adults in rural Kenya and could contribute to evidence-based health policymaking. Our study underscores the relevance of considering socio-demographic factors when making evidence-based policy decisions and designing intervention strategies tailored to the adult population in rural Kenya.

## Supplementary Information

Supplementary Material 1.

## Data Availability

The datasets generated and analysed during the current study are not publicly available in order to protect the anonymity of the participants, but they are available from the corresponding author on reasonable request.
