# Supplementary material for: A cross-sectional study on socio-demographic correlates of self-reported self-care practices for hypertension and type 2 diabetes among adults living in rural Kenya
Source: BMC Health Serv Res. 2024 Dec 19;24:1624. doi: 10.1186/s12913-024-12088-4 (PMC11660613; doi:10.1186/s12913-024-12088-4)
Supplement: Supplementary file 1 — Supplementary Material 1. [file 12913_2024_12088_MOESM1_ESM.docx]

HOUSEHOLD QUESTIONNAIRE

Date of interview: [d][d]/[m][m]/[y][y][y][y] Questionnaire No: [ ][ ][ ]

Research Assistant Name in Full: ­­­­­­­­­­­­­­­­­­­­­­­­­­­­­­­­­­……………………….. Code: …………………….

This is a household questionnaire for a Baseline assessment of the *Prevention and control non communicable diseases (Diabetes & Hypertension) in Imenti South Sub County, Meru County.* The intended goal is to contribute to prevention and control of NCDs by promotion of Healthy Lifestyles. The project is funded by the Danish Red Cross and implemented by the Kenya Red Cross Society (KRCS) and County Government of Meru. The study is being done by researchers from the Reinit Reserch Limited on behalf of KRCS. Findings from this study shall be analysed and compiled into a report for presentation to KRCS and the project donors and shall be used to both measure the success of the current project as well as improve the design of follow up projects. Questions regarding to your demographic information as well as your knowledge and practices around NCDs will be asked using standardized tools. Once again we want to remind you that some of the questions in this interview may be difficult answer or remember. Try to give your best answers and be as honest as possible.

**Ward**

[ ] Aboget

[ ] Igoji

[ ] Igoki

[ ] Kanyakine

[ ] Mikumbune

[ ] Mitiine

[ ] Mitunguu

[ ] Nkuene

**The Person being interviewed is**

[ ] Household Head

[ ] Spouse

**SECTION 1: DEMOGRAPHIC DATA OF THE RESPONDENT**

| *Interviewer: “Now I would like to begin by asking you about your background...”* | | |
| --- | --- | --- |
|  | Sex | [1] Male  [2] Female |
|  | Highest level of Educational attainment | [1] None  [2] Primary  [3] Secondary  [4] Tertiary  [5] Others, Specify: _________ |
|  | Marital Status | [1] Single  [2] Separated  [3] Widowed  [4] Married/living together with a partner (s) as if married  [5] Divorced |
|  | Religious Affiliation | [1] Christian (Protestant)  [2] Christian Catholic  [3] Muslim [4] No religion  [6] Other *(specify……………………* |
|  | What is your household size? | [ ][ ] |
|  | What is your age (in years) | [ ][ ][ ] |
|  | What is your MAIN Source of household income *(one answer only)* | [1] Formal employment  [2] Informal employment  [3] Business  [4] Remittances from kin  [5] Farming  [6] Others, Specify …………. |
|  | What Sector do you work in? | [1] Agriculture  [2] Accounts  [3] Banking or Finance  [4]Building,Construction,Property  [5] Business Management  [6] Creative artisan/Jua Kali  [7] Education  [9] Engineering, Manufacturing, Mining  [10] Enviroment  [11] Healthcare, science and pharmaceutical  [12] Hospitality  [13] Energy and utilities (gas,oil, petrol,waste mangement, water industry)  [14] Information technology, Media  [15] Law, Law enforcement/Security  [16] Lesiure, Sports and Tourism  [17] Marketing, Advertising, PR, Sales  [18] Transport  [19] Other (Specify)­_____________ |
|  | What is YOUR average monthly Income (in *Ksh)* |  |
|  | What is YOUR average monthly expenditure (in *Ksh)* |  |
|  | *In the last 12 months*, on how many separate occasions have you traveled away from your current place of residence and slept away? | [1] None  [2] Greater than zero - record number here [ ][ ][ ]  [3] Don’t know |

**SECTION 2: MEDICAL HISTORY**

| 1. Have you ever been told you suffer from any of the following conditions?  \|  \| Yes \| No \| D/K \| \| --- \| --- \| --- \| --- \| \| High Cholesterol \| ☐ \| ☐ \| ☐ \| \| Diabetes \| ☐ \| ☐ \| ☐ \| \| High blood pressure \| ☐ \| ☐ \| ☐ \| \| Stroke \| ☐ \| ☐ \| ☐ \| \| Cancer of Breast \| ☐ \| ☐ \| ☐ \| \| Cancer of Prostate \| ☐ \| ☐ \| ☐ \| \| Cancer of Cervix \| ☐ \| ☐ \| ☐ \| \| Cancer of Esophagus \| ☐ \| ☐ \| ☐ \| \| Myocardial infarction (Heart Attack) \| ☐ \| ☐ \| ☐ \| \| Poor blood Circulation in the feet \| ☐ \| ☐ \| ☐ \| \| Heart failure \| ☐ \| ☐ \| ☐ \| \| Kidney Disease \| ☐ \| ☐ \| ☐ \| \| Other: \| ☐ \| ☐ \| ☐ \| |  |
| --- | --- | --- | --- | --- | --- | --- | --- | --- | --- | --- | --- | --- | --- | --- | --- | --- | --- | --- | --- | --- | --- | --- | --- | --- | --- | --- | --- | --- | --- | --- | --- | --- | --- | --- | --- | --- | --- | --- | --- | --- | --- | --- | --- | --- | --- | --- | --- | --- | --- | --- | --- | --- | --- | --- | --- | --- | --- |
| 1. If yes in the question above, are you currently taking any of the following?  \|  \| Yes \| No \| Decline \| \| --- \| --- \| --- \| --- \| \| Lipid lowering drugs \| ☐ \| ☐ \| ☐ \| \| Blood pressure drugs \| ☐ \| ☐ \| ☐ \| \| Diabetes drugs \| ☐ \| ☐ \| ☐ \| \| Aspirin \| ☐ \| ☐ \| ☐ \| \| Cancer medicine \| ☐ \| ☐ \| ☐ \| \| Herbal Medicine \| ☐ \| ☐ \| ☐ \| \| Pain Killers \| ☐ \| ☐ \| ☐ \| \| Others \| ☐ \| ☐ \| ☐ \| |  |
| 1. When you are unwell, where do you go for treatment?   ☐ Dispensary  ☐ Health Centre  ☐ County Hospital  ☐ Private Hospital/Clinic  ☐ Herbalist  ☐ Mission Hospital  ☐ Private Chemist  ☐ Spiritual Healer  ☐ Sub-county hospital  ☐ Laboratory  ☐ National Referral hospital |  |
| 1. Do you have any history of any of the following in your family?  \|  \| Yes \| No \| D/K \| \| --- \| --- \| --- \| --- \| \| High Cholesterol \| ☐ \| ☐ \| ☐ \| \| Diabetes \| ☐ \| ☐ \| ☐ \| \| High blood pressure \| ☐ \| ☐ \| ☐ \| \| Prostate Cancer \| ☐ \| ☐ \| ☐ \| \| Breast Cancer \| ☐ \| ☐ \| ☐ \| \| Cervical Cancer \| ☐ \| ☐ \| ☐ \| \| Heart attack \| ☐ \| ☐ \| ☐ \| \| Stroke \| ☐ \| ☐ \| ☐ \| \| Treatment for mental illness \| ☐ \| ☐ \| ☐ \| |  |
| 1. For female respondents; _ Are you on any family planning method?   ◻Yes  ◻ Never  ◻ Stopped   1. If yes or stopped; What method of family planning do you currently /did you use?   □ Condoms  □ Sterilization/Hysterectomy  □ Intrauterine Device  □ Norplant  □ Diaphragm/Cervical Cap  □ Oral Contraceptive Pills  □ Natural Family Planning/Rhythm  □ Injectable Hormones  □ Don’t Know  □ None  □ Other Specific | If YES Skip to 17 |

**SECTION 3: KNOWLEDGE LEVELS, ATTITUDE AND PRACTICES TOWARD NCDS**

| The next few questions will help us to evaluate your knowledge of these select non communicable diseases; hypertension & diabetes   1. How do you rate your knowledge on these diseases?  \| ☐   Very well informed \| ☐   Well informed \| ☐   Moderately informed \| \| --- \| --- \| --- \| \| ☐   Not at all informed \| ☐   Do not know/No answer \| \|  1. Have you ever had a discussion with your doctor/health care worker on these diseases when talking about your health?   ◻   Yes  ◻  No  ◻   Don’t know |
| --- | --- | --- | --- | --- | --- | --- |
| 1. During the last three years has a doctor or other health worker advised you to do any of the following?  \|  \| Yes \| No \| N/A \| \| --- \| --- \| --- \| --- \| \| Quit using tobacco or do not start \| ☐ \| ☐ \| ☐ \| \| Reduce, stop or do not start using alcohol \| ☐ \| ☐ \| ☐ \| \| Reduce salt in your diet \| ☐ \| ☐ \| ☐ \| \| Eat at least five servings of fruit a day \| ☐ \| ☐ \| ☐ \| \| Reduce fat in your diet \| ☐ \| ☐ \| ☐ \| \| Start or do more physical activity \| ☐ \| ☐ \| ☐ \| \| Maintain a healthy body weight or lose weight \| ☐ \| ☐ \| ☐ \| \| Do self-breast examination regularly \| ☐ \| ☐ \| ☐ \| \| Attend cervical cancer screening \| ☐ \| ☐ \| ☐ \|  1. Are you comfortable talking with your Physician about preventive health options and treatment options regarding your health?   ◻   Yes  ◻ Somewhat comfortable  ◻   Not comfortable  ◻   Do Not Know   1. If not comfortable, why?   ◻   No time  ◻ Clinician busy  ◻ Afraid of clinician  ◻  Find no reason to discuss   1. Within the past year, have you ever seen, heard, or read information about these diseases?   ◻ Yes  ◻ No     1. If answer is Yes, which disease?   ☐ Diabetes  ☐Hypertension  ☐ Breast cancer  ☐Cervical cancer   1. If answer is Yes, what was your source of information  \| ☐ Radio \| ☐ Television \| ☐Magazine/news-papers \| \| --- \| --- \| --- \| \| ☐Text message \| ☐ Family/friends \| ☐Health care professional \| \| ☐ Brochure/books \| ☐ Internet \| ☐ Community Health volunteer \| \| ☐ Other sources \| ☐ Community meetings \| ☐ Don’t  know \| |
|  |
| Now I would like you to tell me all the causes/risk factors of these non-communicable diseases (o*pen ended question)*   \| 1. Diabetes \| \| \| \| --- \| --- \| --- \| \| ☐ High blood sugar \| ☐Overweight/obese \| ☐Advanced age \| \| ☐ High blood pressure \| ☐Stress \| ☐Curse/witchcraft \| \| ☐ High cholesterol \| ☐Smoking/Tobacco use \| ☐ Don’t know \| \| ☐ Physical inactivity \| ☐Inherited risk \| ☐ Other : ________________ \| \| ☐ Drinking alcohol \| ☐High salt Intake \|  \|  \| 1. Hypertension \| \| \| \| --- \| --- \| --- \| \| ☐ High blood sugar \| ☐Overweight/obese \| ☐Advanced age \| \| ☐ High blood pressure \| ☐Stress \| ☐Curse/witchcraft \| \| ☐ High cholesterol \| ☐Smoking/Tobacco use \| ☐ Don’t know \| \| ☐ Physical inactivity \| ☐Inherited risk \| ☐ Other : ________________ \| \| ☐ Drinking alcohol \| ☐High salt Intake \| ☐ \| |
|  |
| \| 1. Can you tell me of all the signs of diabetes? *(Open-ended questions)* \| 1. Can you tell me of all the signs of hypertension? *(Open-ended questions)* \| \| --- \| --- \| \| ☐ Increased thirst \| ☐ Severe headache \| \| ☐ Increased hunger, even after eating \| ☐ Fatigue \| \| ☐ Frequent urination \| ☐ Loss of consciousness \| \| ☐ Unexplained weight loss \| ☐ Confusion \| \| ☐ Extreme Fatigue/tired feeling \| ☐ Chest pain \| \| ☐ Blurred vision \| ☐ Difficulty breathing \| \| ☐ Headache \| ☐ Irregular heartbeat \| \| ☐ Cuts/bruises healing slowly \| ☐ Nose bleeding \| \| ☐ Tingling pain or numbness in hands/feet \| ☐ Pounding in the chest, neck or ears \| \| ☐ I don’t know \| ☐ I don’t know \| \| ☐ Others: __________________ \| ☐ Others: _____________________ \| \|  \|  \| |
|  |
| 1. What is heart attack?   ☐ Sudden stopping of heart beat.  ☐ Sudden death.  ☐ Death of a section of the heart muscle due to blocked blood supply.  ☐ Sudden collapse  ☐ Don’t know  ☐ Other (specify)__________ |
|  |
| \| 1. Can you tell me of all the warning signs of heart attack? *(Open-ended question)* \| 1. Now I would like you to tell me the causes/risk factors of heart attack about which you now *(Open-ended question)* \| \| --- \| --- \| \| ☐ Difficulty breathing \| ☐ High blood sugar \| \| ☐ Dizziness or light headedness \| ☐ High blood pressure \| \| ☐ Loss of consciousness \| ☐ High cholesterol \| \| ☐ Back pain \| ☐ Physical inactivity \| \| ☐ Pain in the chest with/without exercise \| ☐ Drinking alcohol \| \| ☐ Excessive sweating \| ☐ Overweight/Obese \| \| ☐ Vomiting tendency \| ☐ Stress \| \| ☐ Pain in the teeth or jaw \| ☐ Smoking/Tobacco use \| \| ☐ Pain in the abdomen \| ☐ Inherited risk \| \| ☐ I don’t know \| ☐ Age \| \| ☐ Others: ________________________ \| ☐ Other \| \|  \| ☐ Do not know answer/No answer \| |
|  |
| 1. If someone gets a heart attack at home what will you do or won’t do?  \|  \| Will do \| Won’t do \| Not Sure \| \| --- \| --- \| --- \| --- \| \| Treat at home \| ☐ \| ☐ \| ☐ \| \| Immediately take the person to the hospital \| ☐ \| ☐ \| ☐ \| \| Take to a pharmacy/medicine shop \| ☐ \| ☐ \| ☐ \| \| Wait for appointment with a heart specialist \| ☐ \| ☐ \| ☐ \| \| Take the patient to a traditional healer \| ☐ \| ☐ \| ☐ \| \| Give home-made therapy \| ☐ \| ☐ \| ☐ \| \| Don’t know \| ☐ \| ☐ \| ☐ \| \| Other \| ☐ \| ☐ \| ☐ \| |
|  |
| 1. What is stroke?   ☐ Sudden weakness of one side of the body  ☐ Sudden collapse  ☐ Sudden death  ☐ Death of a part of the brain due to blocked blood supply to it.  ☐ Don’t Know  ☐ Other |
|  |
| 1. Now I would like you to tell me all the causes/risk factors of Stroke about which you know *(Open-ended question)*  \| ☐ High blood pressure \| ☐ High cholesterol \| ☐ Inherited \| \| --- \| --- \| --- \| \| ☐ Diabetes \| ☐ Cigarette smoking \| ☐ Don’t know \| \| ☐ Overweight \| ☐ Alcohol use \| ☐ Others: \| \| ☐ Physical inactivity \| ☐ Stress \|  \| |
|  |
| 1. Attitude towards cardiovascular and diabetes risk factor modification  \| Attitude Question towards risk factor modification \| Agree \| Disagree \| Don’t Know \| N/A \| \| --- \| --- \| --- \| --- \| --- \| \| Diabetes  can be prevented \| ☐ \| ☐ \| ☐ \| ☐ \| \| CVD diseases such as stroke and heart diseases can be prevented \| ☐ \| ☐ \| ☐ \| ☐ \| \| I am at risk for  diabetes, \| ☐ \| ☐ \| ☐ \| ☐ \| \| I am at risk for heart diseases or stroke. \| ☐ \| ☐ \| ☐ \| ☐ \| \| Cancers such as cervical and breast  can be prevented \| ☐ \| ☐ \| ☐ \| ☐ \| \| I am at risk of  cancer \| ☐ \| ☐ \| ☐ \| ☐ \| \| My present weight is too high for my health \| ☐ \| ☐ \| ☐ \| ☐ \| \| The food I eat is low in vegetables and fruits, and I would like to balance it. \| ☐ \| ☐ \| ☐ \| ☐ \| \| Changing my behavior such as smoking, and excessive drinking of alcohol will reduce my risk of developing diabetes and heart disease. \| ☐ \| ☐ \| ☐ \| ☐ \| \| Changing my behavior such as smoking, and excessive drinking of alcohol will reduce my risk of developing cancer. \| ☐ \| ☐ \| ☐ \| ☐ \| \| Reducing salt intake will reduce my risk of developing hypertension and heart diseases. \| ☐ \| ☐ \| ☐ \| ☐ \| \| Increasing physical activity will reduce my risk of developing diabetes and heart diseases. \| ☐ \| ☐ \| ☐ \| ☐ \| \| I don’t have time to adopt healthy behavior (exercise, prepare healthy meals, go for health screenings) \| ☐ \| ☐ \| ☐ \| ☐ \| \| Herbal medicines are easier and more useful than lifestyle changes in preventing and controlling diabetes and CVD. \| ☐ \| ☐ \| ☐ \| ☐ \| \| God or a higher power ultimately determines my health, not me. \| ☐ \| ☐ \| ☐ \| ☐ \| \| I would go to hospital for checkups when I am not sick to prevent ailments. \| ☐ \| ☐ \| ☐ \| ☐ \| |
|  |
| 1. This section will tell us more about your current practices towards better health.   In the past 3 years,   \|  \| Yes \| No \| Not sure \| N/A \| \| --- \| --- \| --- \| --- \| --- \| \| Have you had your blood pressure measured? \| ☐ \| ☐ \| ☐ \|  \| \| Have you had your sugar checked? \| ☐ \| ☐ \| ☐ \|  \| \| Have you had your cholesterol checked? \| ☐ \| ☐ \| ☐ \|  \| \| Have you had a self-breast examination? \| ☐ \| ☐ \| ☐ \|  \| \| Have you had screening for cervical cancer? \| ☐ \| ☐ \| ☐ \|  \| \| Have you tried to increase the amount of fruits and vegetables you eat? \| ☐ \| ☐ \| ☐ \|  \| \| Have you tried to quit smoking? \| ☐ \| ☐ \| ☐ \|  \| \| Have you tried to cut weight? \| ☐ \| ☐ \| ☐ \|  \| \| Have you tried to increase your physical activity? \| ☐ \| ☐ \| ☐ \|  \| \| Have you tried to reduce/ stop using alcohol? \| ☐ \| ☐ \| ☐ \|  \| |
|  |
| 1. What was the reason for getting the above tests/change your behavior (perceived benefits)   ☐ Wanted to feel better  ☐ I did it for the family  ☐ Wanting to avoid medications  ☐ Advised by a health professional/  ☐ Worried about getting heart diseases because a friend/family member died of it  ☐ other ____________ |
|  |

**SECTION 4: NCD RISK FACTORS**

**Social history**

| A. Tobacco   1. Do you use tobacco?   ◻Yes  ◻ Never  ◻ Stopped  ◻ Refuse to answer   1. If yes, how long have you been using tobacco?   ________ Years. 2. If you use tobacco or used to use tobacco, what kind do (did) you usually   use   (tick all that applies)?  ◻ Cigarette  ◻ Pipe  ◻ Chew  ◻ Cigar  ◻ Snuff  ◻ Other:   1. If yes, how many sticks per day?   ◻   1-5  ◻   6-10  ◻   11-20  ◻   >20   1. If you quit using tobacco, when did you last use it?   ◻ Less than 12 months ago  ◻ More than 12 months ago |
| --- |
| **Alcohol**   1. Have you ever consumed any alcohol?   ◻   Yes  ◻ Never  ◻ Stopped  ◻ Refuse   1. If yes or stopped, have you consumed any alcohol in the last 12 months? ◻   Yes          ◻ No 2. If yes, how frequently did you take alcohol?   ◻ Daily  ◻5-6 days per week  ◻ 3-4 days per week  ◻1-2 days per week  ◻ 1-3 days per month     1. If you drink alcohol or used to drink alcohol, what kind do (did) you usually drink? *(Tick all that apply)*   ◻ Chang’aa  ◻Spirits  ◻ Beer  ◻Wine  ◻ Busaa  ◻ Others   1. How many drinks containing alcohol did you have on a typical day when you were drinking alcohol in the past year?   ◻ 1 to 2 drinks  ◻ 3 to 4 drinks  ◻ 5 to 6 drinks  ◻ 7 to 9 drinks  ◻ 10 or more drinks   1. How often did you have six or more drinks on one occasion in the past year?   ◻ Never  ◻ Less than monthly  ◻ Monthly  ◻ Weekly  ◻ Daily or almost daily |
| **PHYSICAL ACTIVITY** |
| **Activities at Work**  I am going to ask you about the time you spend doing different types of physical activity in a typical week. Please answer these questions even if you do not consider yourself to be a physically active person. Think first about the time you spend doing work.  Think of work as the things that you have to do such as paid or unpaid work, study/training, household chores, harvesting food/crops, fishing or seeking   employment. In answering the following questions 'vigorous-intensity activities' are activities that require hard physical effort and cause large increases in breathing or heart rate, 'moderate-intensity activities are those that require moderate physical effort and cause small increases in breathing/ heart rate. |
| 1. Does your work involve vigorous-intensity activity that causes large increases in breathing or heart rate? *[heavy lifting, running, playing football, fast cycling, oxen ploughing, cart pulling, digging and boat rowing]?*◻   Yes           ◻   No 2. If *yes*, how much time do you spend doing vigorous-intensity activity on a given day? _____Hrs ____min 3. In a typical week, on how many days do you do vigorous-intensity activities?   As part of your work?   ________days |
| 1. Does your work involve moderate-intensity activity that require moderate physical effort and cause small increases in breathing/ heart rate (brisk walking, carrying light loads, cycling at a regular pace, manual washing, fetching water from a well or milking clothes? Do not include walking unless you are purposely walking at a fast pace )◻   Yes           ◻   No 2. If *yes*, how much time do you spend doing moderate-intensity activity on a given day? _____Hrs ____min 3. In a typical week, on how many days do you do moderate-intensity activities?   As part of your work?   ________days |
| **Travel to and from places**  The next questions exclude the physical activities at work that you have already mentioned. Now I would like to ask you about the usual way you travel/move to and from places. For example to work, for shopping, to the market, to place of worship, to visit friends and relatives   1. Do you walk or ride a bicycle yourself for at least 10 minutes continuously to get to and from places?   **◻ Yes**  **◻  No**   1. In a typical week, on how many days do you walk or ride a bicycle yourself for at least 10 minutes continuously to get to and from places? ______ Days. 2. How much time do you spend walking or cycling on a typical day? _______ Hours. |
| **Recreational activities**  The next questions exclude the work and transport activities that you have already mentioned. Now I would like to ask you about sports, fitness and recreational activities (leisure).   1. Do you do any vigorous-intensity sports, fitness or recreational   (leisure) activities that cause large increases in breathing or heart rate like [treadmill, running, football, volleyball, skipping rope, aerobic dancing, hiking up hill, jogging ] for at least 10 minutes continuously  ◻   Yes  ◻   No   1. In a typical week, on how many days do you do vigorous-intensity sports, fitness or recreational (leisure) activities? ______ Days 2. How much time do you spend doing vigorous-intensity sports, fitness or recreational activities on a typical day? ______ Hours 3. Do you do any moderate-intensity sports, fitness or recreational (leisure) activities that cause a small increase in breathing/heart rate like [casual walk, dancing, cycling or swimming] for at least 10 min. continuously ◻        Yes        ◻    No 4. In a typical week, on how many days do you do moderate- intensity sports, fitness or recreational (leisure) activities? _____ Days 5. How much time do you spend doing moderate-intensity sports, fitness or recreational activities on a typical day? ______ Hours |
| **Sedentary behavior**  The following question is about sitting or reclining at work, at home, getting to and from places, or with friends including time spent sitting at a desk, sitting with friends, travelling in car, bus, or matatu, reading, playing cards, watching television, on facebook, Whatsapp, instagram, betting, chamas or jumuia but do not include sleeping].   1. How much time do you usually spend sitting or reclining on a typical day (not including sleeping)? ______ Hours 2. How many hours do you usually spend sleeping in a typical 24 hour day? _____ Hours |
| **24 HOUR FOOD RECALL.**  Now I would like to ask you about food that you ate in the last 24hours. Please list anything that you ate from the time you woke up yesterday until when you went to sleep. It can be a food you ate at home, in a hotel or at a friend’s house. NB: Remember to mention any fruits or drinks that you also took   \| **Cereals:** \| Maize (Uji from Maize, Ugali, boiled maize, roasted maize, in githeri, cornflakes)  Sorghum (Uji/ Ugali)  Millet (Uji/Ugali)  Rice  Wheat (white or brown bread, biscuits, cake, chapati, popcorn, mandazi, kangumu, doughnuts)  None  Others: ______________________________________________________ \| \| --- \| --- \| \| **Roots and Tubers** \| Nduma Sweet potatoes Irish potatoes (chips, potato crisps) Cassava-Muhogo) None Others: ___________________________ \| \| **Vegetables** \| Cabbages Sukuma wiki Spinach Managu  Kunde leaves Mitoo Pumpkin leaves Carrots  None Others: ____________________________________________ \| \| **Fruits** \| Pineapple Watermelon Oranges Ripe bananas Mangoes Quava – mapera Pawpaw Avocado Mfenesi ( Jackfruit) Apples Passion fruit Berries Msabibu Grapes Pear None Others: ______________________________ \| \| **Meat/Poultry/ Ofals** \| Beef Chicken Pork Sheep Goat Duck  Rabbit Pigeons Turkey Guinea fowls Quail Liver Heart Kidneys Tongue of any animal Mutura Matumbo None Others: ____________________________________________ \| \| **Eggs** \| Chicken eggs Quail eggs None Others: ________________ \| \| **Fish/ Seafood** \| Tilapia Omena Nileperch/Mbuta Mudfish- kamongo    Catfish None Others: _________________________________ \| \| **Pulses/ Legumes/ Nuts** \| Peas Lentils Beans-maharagwe Green grams- Ndengu  Njugu_all types None Others: ____________________________ \| \| **Milk and milk products** \| Cheese Yoghurt Fresh Milk Sour Milk/ Mala/Mursik Ghee  Butter Cream None Others: __________________________ \| \| **Oil/ Fats** \| Margarine Cooking oil None Others: ____________________ \| \| **Sugar/Honey** \| Taken in tea Coffee None \| \| **Miscellaneous** \| If there is a food item that the participant ate but you are not sure of the category kindly indicate it here.  _____________________________________________________________ \| \|  \| Were you at a party, wedding, funeral or function yesterday?  Yes No \| |
| **Nutrition /Diet**   1. In a typical week, on how many days do you eat fruit (*like pineapples, oranges, guavas,ripe bananas,plums,berries,pawpaw,Avocado,water melon,mfenesi, loquats,pears, mangos*)?  ________ days (*one number only*) if *zero*, go to vegetables 2. On average, how many servings of fruit(a serving of fruit could be 2 guavas/half an orange/half an Avocado) do you eat on one of those days? __________ servings (*one number only*)      1. In a typical week, on how many days do you eat vegetables (like sukuma wiki, managu, cabbage)?  ________ Days (one number only**)** 2. On average, how many servings of vegetables (A serving of vegetables is 3 heaped table spoons) do you eat on one of those days?  __________ Servings (One number only)      1. How often do you add salt to your food after it is cooked?   ◻   Never  ◻  Often after tasting  ◻   Often before tasting     1. How often is salty seasoning or salty sauce added in cooking or preparingg food in your household?   ◻   Always  ◻  Sometimes  ◻   Never   1. How often do you use processed foods high in salt? (Bread, crisps, salted nuts, bacon, sausages, popcorn, salt preserved meat or fish)   ◻   Always  ◻  Sometimes  ◻   Never   1. What type of oil or fat do you use in your household?   ◻ Vegetable oil  ◻ Solid fat  ◻ Butter/ghee/cream  ◻ Margerine  ◻ Other  ◻ None   1. How many meals per week do you eat that are not prepared at home? ( E.g Eating in restaurants or take away) ______ |
